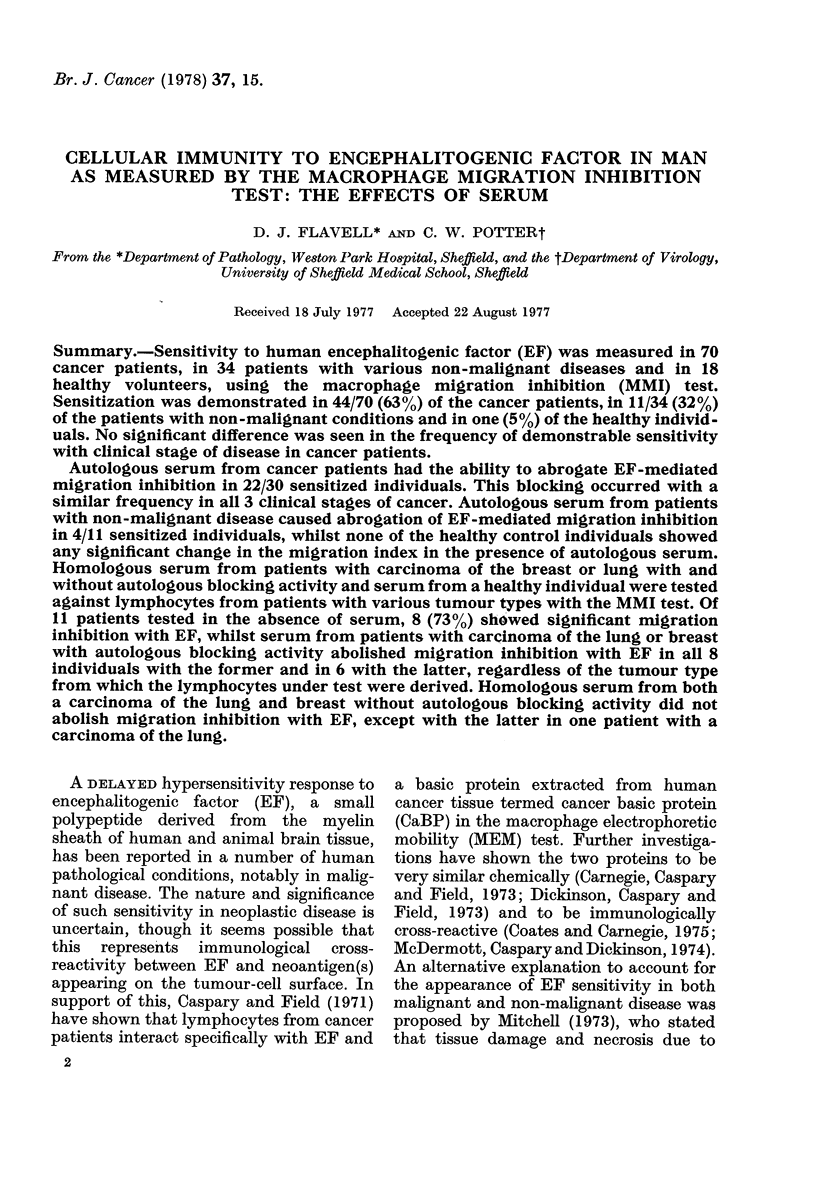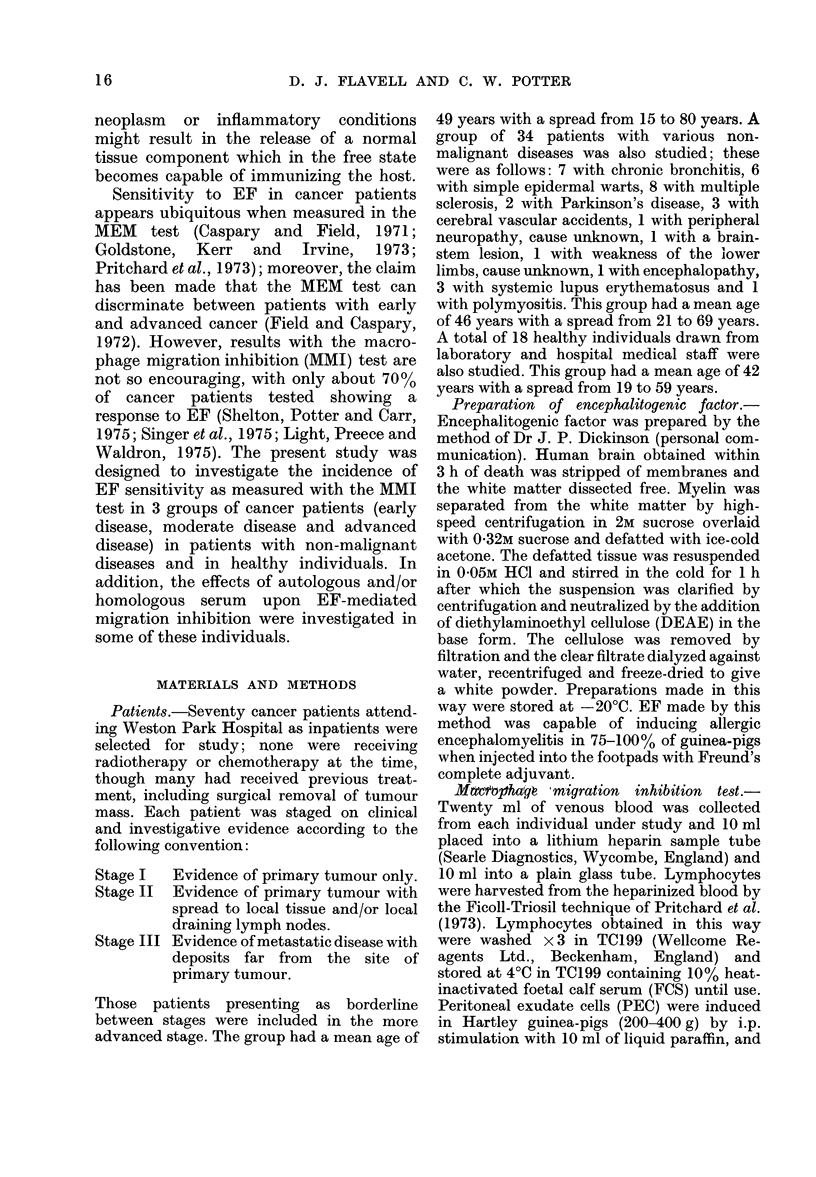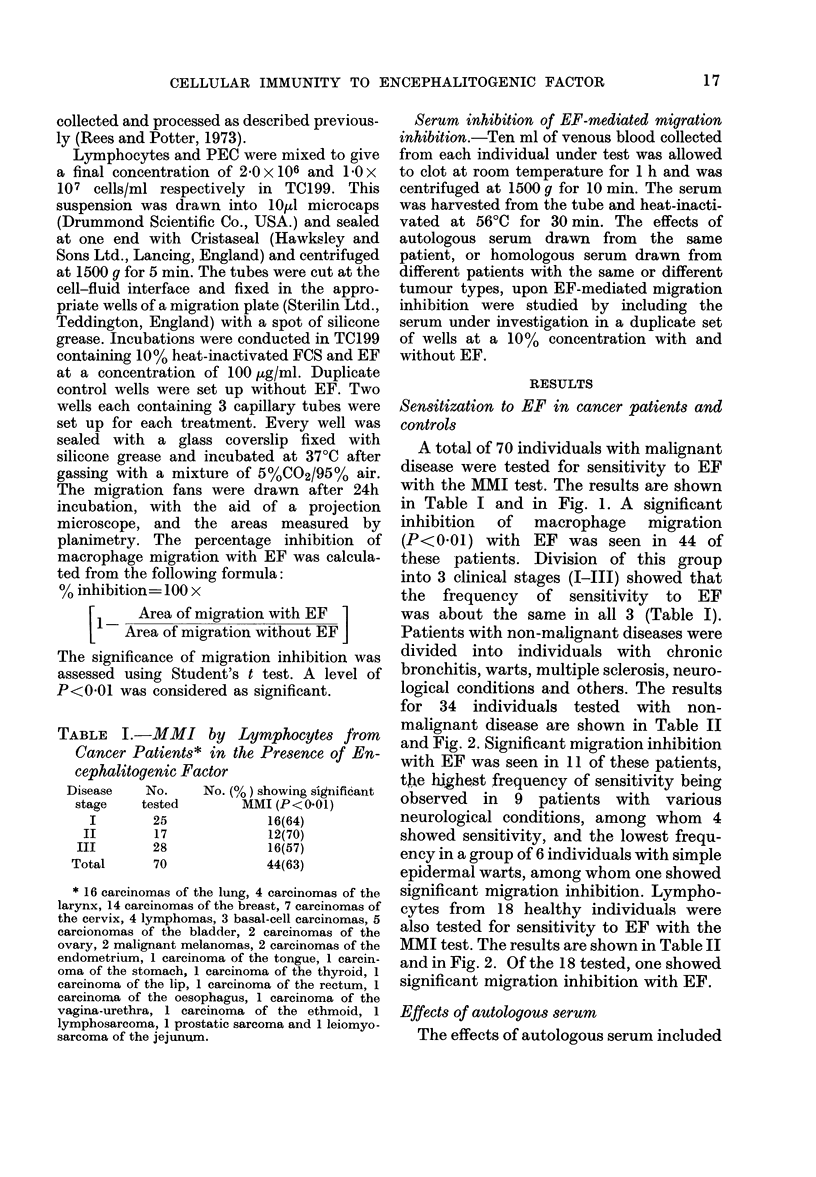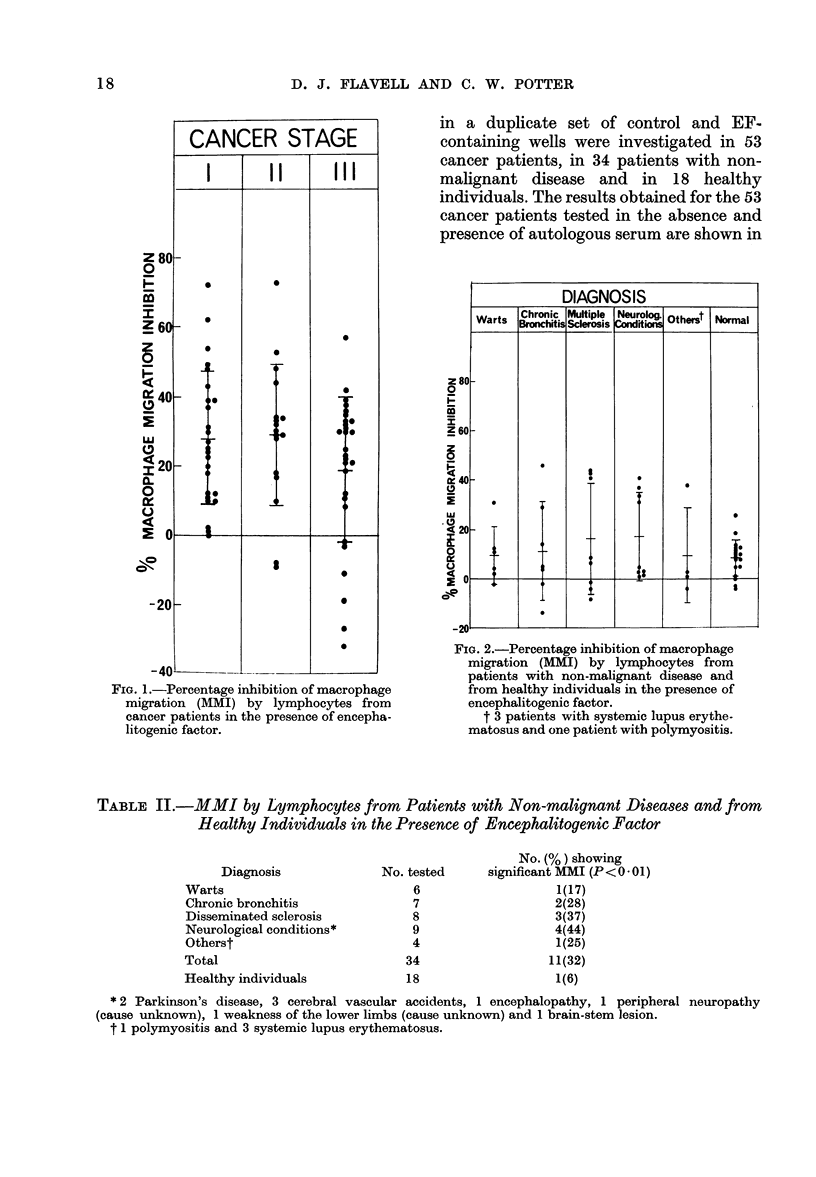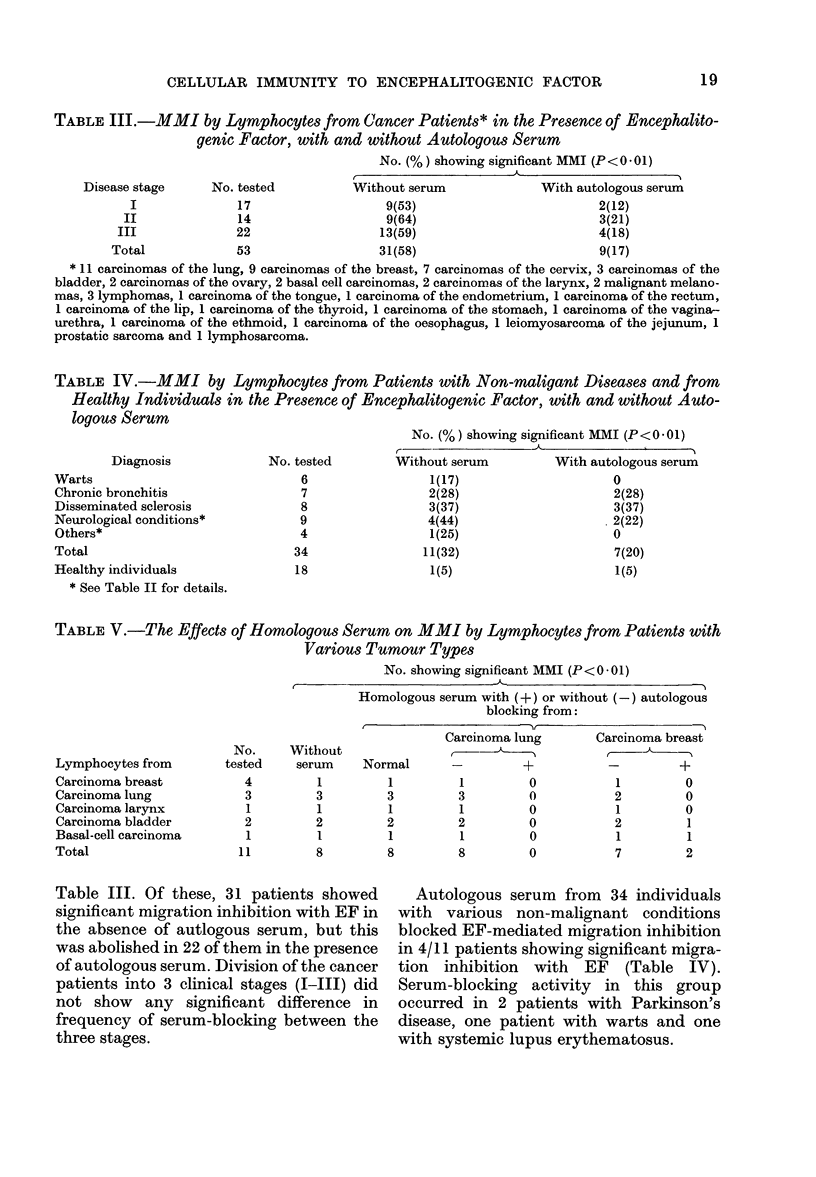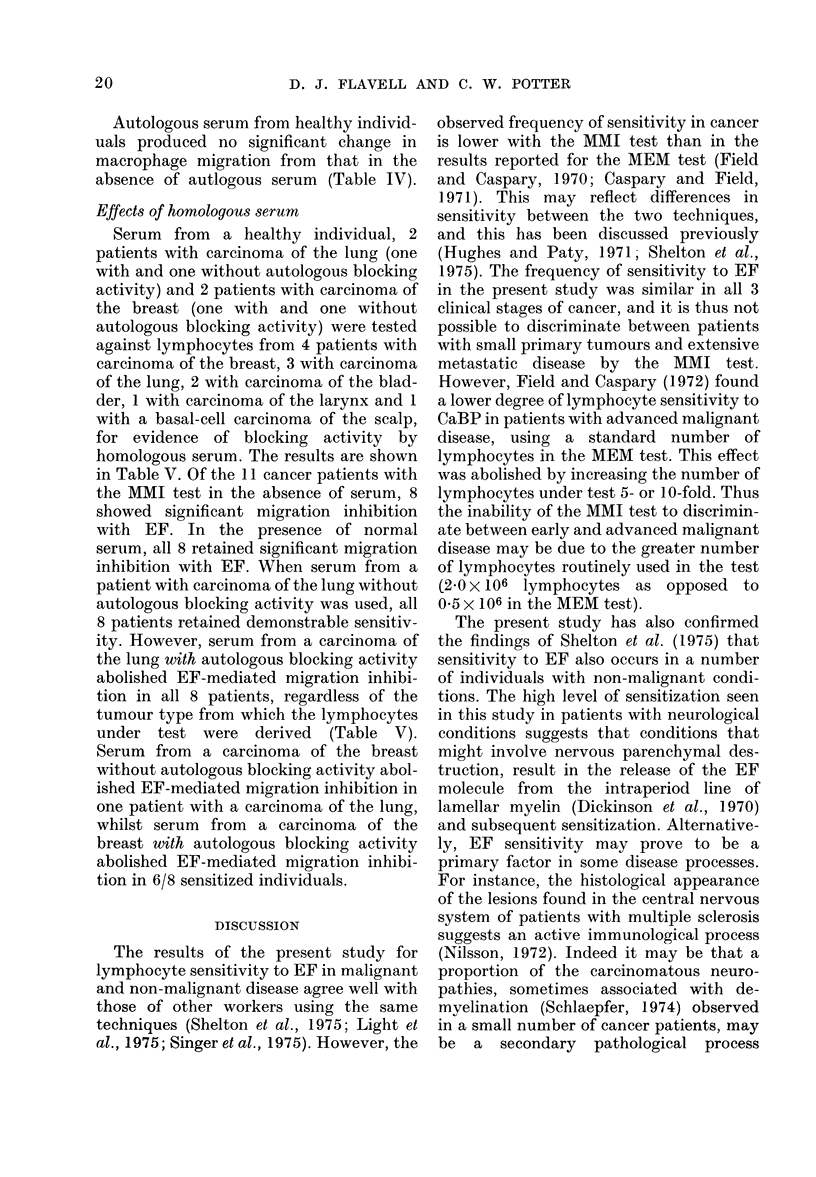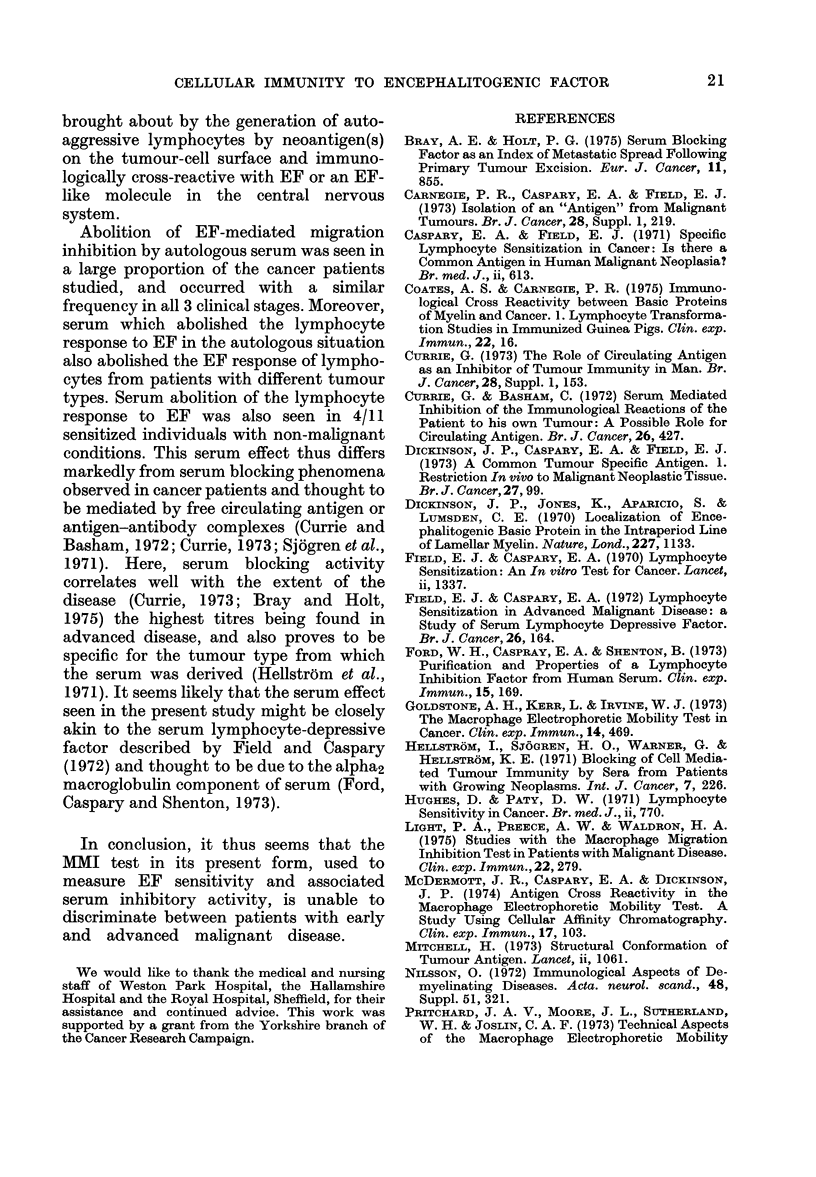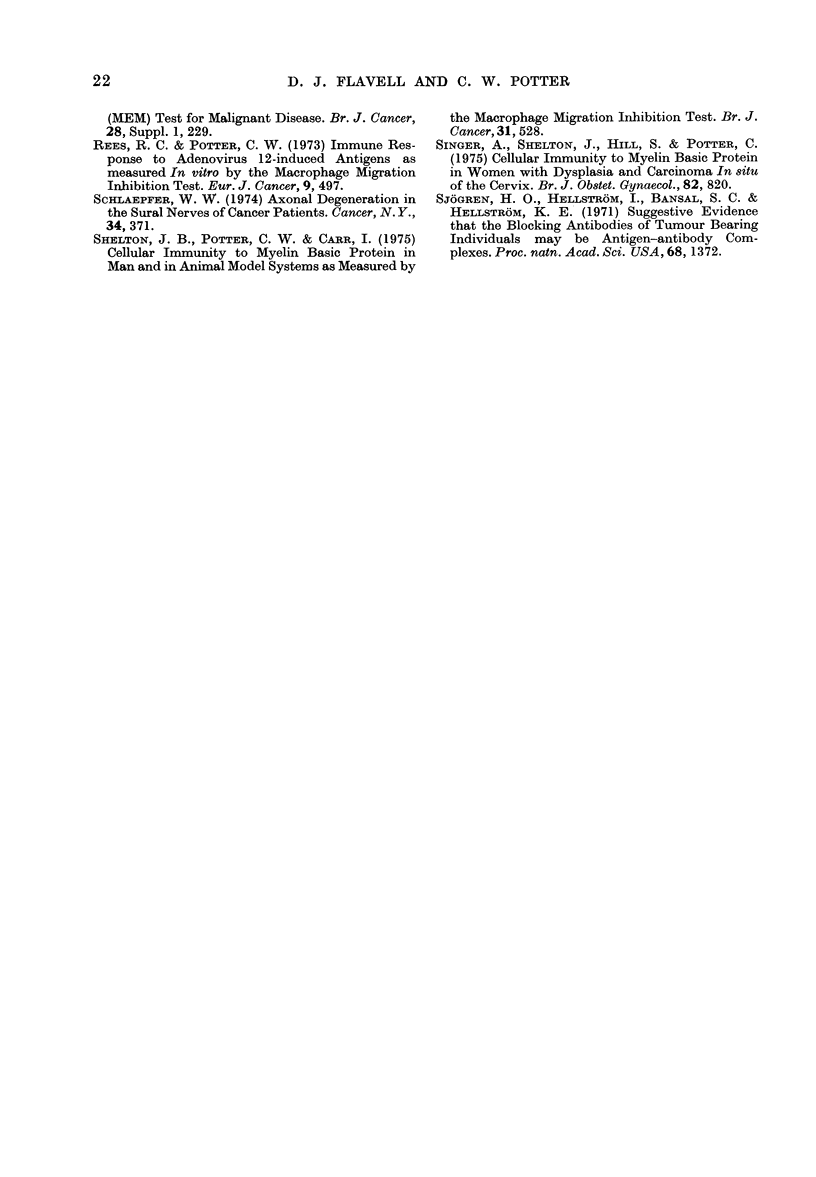# Cellular Immunity to Encephalitogenic Factor in Man as Measured by the Macrophage Migration Inhibition Test: The Effects of Serum

**DOI:** 10.1038/bjc.1978.2

**Published:** 1978-01

**Authors:** D. J. Flavell, C. W. Potter

## Abstract

Sensitivity to human encephalitogenic factor (EF) was measured in 70 cancer patients, in 34 patients with various non-malignant diseases and in 18 healthy volunteers, using the macrophage migration inhibition (MMI) test. Sensitization was demonstrated in 44/70 (63%) of the cancer patients, in 11/34 (32%) of the patients with non-malignant conditions and in one (5%) of the healthy individuals. No significant difference was seen in the frequency of demonstrable sensitivity with clinical stage of disease in cancer patients.

Autologous serum from cancer patients had the ability to abrogate EF-mediated migration inhibition in 22/30 sensitized individuals. This blocking occurred with a similar frequency in all 3 clinical stages of cancer. Autologous serum from patients with non-malignant disease caused abrogation of EF-mediated migration inhibition in 4/11 sensitized individuals, whilst none of the healthy control individuals showed any significant change in the migration index in the presence of autologous serum. Homologous serum from patients with carcinoma of the breast or lung with and without autologous blocking activity and serum from a healthy individual were tested against lymphocytes from patients with various tumour types with the MMI test. Of 11 patients tested in the absence of serum, 8 (73%) showed significant migration inhibition with EF, whilst serum from patients with carcinoma of the lung or breast with autologous blocking activity abolished migration inhibition with EF in all 8 individuals with the former and in 6 with the latter, regardless of the tumour type from which the lymphocytes under test were derived. Homologous serum from both a carcinoma of the lung and breast without autologous blocking activity did not abolish migration inhibition with EF, except with the latter in one patient with a carcinoma of the lung.


*Br. J. Cancer* (1978) **37**, 15.

### CELLULAR IMMUNITY TO ENCEPHALITOGENIC FACTOR IN MAN AS MEASURED BY THE MACROPHAGE MIGRATION INHIBITION TEST: THE EFFECTS OF SERUM

D. J. FLAVELL* AND C. W. POTTER†

*From the *Department of Pathology, Weston Park Hospital, Sheffield, and the †Department of Virology, University of Sheffield Medical School, Sheffield*

Received 18 July 1977 Accepted 22 August 1977

**Summary.—Sensitivity to human encephalitogenic factor (EF) was measured in 70 cancer patients, in 34 patients with various non-malignant diseases and in 18 healthy volunteers, using the macrophage migration inhibition (MMI) test. Sensitization was demonstrated in 44/70 (63%) of the cancer patients, in 11/34 (32%) of the patients with non-malignant conditions and in one (5%) of the healthy individuals. No significant difference was seen in the frequency of demonstrable sensitivity with clinical stage of disease in cancer patients.**

**Autologous serum from cancer patients had the ability to abrogate EF-mediated migration inhibition in 22/30 sensitized individuals. This blocking occurred with a similar frequency in all 3 clinical stages of cancer. Autologous serum from patients with non-malignant disease caused abrogation of EF-mediated migration inhibition in 4/11 sensitized individuals, whilst none of the healthy control individuals showed any significant change in the migration index in the presence of autologous serum. Homologous serum from patients with carcinoma of the breast or lung with and without autologous blocking activity and serum from a healthy individual were tested against lymphocytes from patients with various tumour types with the MMI test. Of 11 patients tested in the absence of serum, 8 (73%) showed significant migration inhibition with EF, whilst serum from patients with carcinoma of the lung or breast with autologous blocking activity abolished migration inhibition with EF in all 8 individuals with the former and in 6 with the latter, regardless of the tumour type from which the lymphocytes under test were derived. Homologous serum from both a carcinoma of the lung and breast without autologous blocking activity did not abolish migration inhibition with EF, except with the latter in one patient with a carcinoma of the lung.**

A DELAYED hypersensitivity response to encephalitogenic factor (EF), a small polypeptide derived from the myelin sheath of human and animal brain tissue, has been reported in a number of human pathological conditions, notably in malignant disease. The nature and significance of such sensitivity in neoplastic disease is uncertain, though it seems possible that this represents immunological cross-reactivity between EF and neoantigen(s) appearing on the tumour-cell surface. In support of this, Caspary and Field (1971) have shown that lymphocytes from cancer patients interact specifically with EF and a basic protein extracted from human cancer tissue termed cancer basic protein (CaBP) in the macrophage electrophoretic mobility (MEM) test. Further investigations have shown the two proteins to be very similar chemically (Carnegie, Caspary and Field, 1973; Dickinson, Caspary and Field, 1973) and to be immunologically cross-reactive (Coates and Carnegie, 1975; McDermott, Caspary and Dickinson, 1974). An alternative explanation to account for the appearance of EF sensitivity in both malignant and non-malignant disease was proposed by Mitchell (1973), who stated that tissue damage and necrosis due to neoplasm or inflammatory conditions might result in the release of a normal tissue component which in the free state becomes capable of immunizing the host.

Sensitivity to EF in cancer patients appears ubiquitous when measured in the MEM test (Caspary and Field, 1971; Goldstone, Kerr and Irvine, 1973; Pritchard *et al.*, 1973); moreover, the claim has been made that the MEM test can discrminate between patients with early and advanced cancer (Field and Caspary, 1972). However, results with the macrophage migration inhibition (MMI) test are not so encouraging, with only about 70% of cancer patients tested showing a response to EF (Shelton, Potter and Carr, 1975; Singer *et al.*, 1975; Light, Preece and Waldron, 1975). The present study was designed to investigate the incidence of EF sensitivity as measured with the MMI test in 3 groups of cancer patients (early disease, moderate disease and advanced disease) in patients with non-malignant diseases and in healthy individuals. In addition, the effects of autologous and/or homologous serum upon EF-mediated migration inhibition were investigated in some of these individuals.

### MATERIALS AND METHODS

*Patients.*—Seventy cancer patients attending Weston Park Hospital as inpatients were selected for study; none were receiving radiotherapy or chemotherapy at the time, though many had received previous treatment, including surgical removal of tumour mass. Each patient was staged on clinical and investigative evidence according to the following convention:

| | |
|---|---|
| Stage I | Evidence of primary tumour only. |
| Stage II | Evidence of primary tumour with spread to local tissue and/or local draining lymph nodes. |
| Stage III | Evidence of metastatic disease with deposits far from the site of primary tumour. |

Those patients presenting as borderline between stages were included in the more advanced stage. The group had a mean age of 49 years with a spread from 15 to 80 years. A group of 34 patients with various non-malignant diseases was also studied; these were as follows: 7 with chronic bronchitis, 6 with simple epidermal warts, 8 with multiple sclerosis, 2 with Parkinson's disease, 3 with cerebral vascular accidents, 1 with peripheral neuropathy, cause unknown, 1 with a brain-stem lesion, 1 with weakness of the lower limbs, cause unknown, 1 with encephalopathy, 3 with systemic lupus erythematosus and 1 with polymyositis. This group had a mean age of 46 years with a spread from 21 to 69 years. A total of 18 healthy individuals drawn from laboratory and hospital medical staff were also studied. This group had a mean age of 42 years with a spread from 19 to 59 years.

*Preparation of encephalitogenic factor.*—Encephalitogenic factor was prepared by the method of Dr J. P. Dickinson (personal communication). Human brain obtained within 3 h of death was stripped of membranes and the white matter dissected free. Myelin was separated from the white matter by high-speed centrifugation in 2M sucrose overlaid with 0·32M sucrose and defatted with ice-cold acetone. The defatted tissue was resuspended in 0·05M HCl and stirred in the cold for 1 h after which the suspension was clarified by centrifugation and neutralized by the addition of diethylaminoethyl cellulose (DEAE) in the base form. The cellulose was removed by filtration and the clear filtrate dialyzed against water, recentrifuged and freeze-dried to give a white powder. Preparations made in this way were stored at −20°C. EF made by this method was capable of inducing allergic encephalomyelitis in 75–100% of guinea-pigs when injected into the footpads with Freund's complete adjuvant.

*Macrophage migration inhibition test.*—Twenty ml of venous blood was collected from each individual under study and 10 ml placed into a lithium heparin sample tube (Searle Diagnostics, Wycombe, England) and 10 ml into a plain glass tube. Lymphocytes were harvested from the heparinized blood by the Ficoll-Triosil technique of Pritchard *et al.* (1973). Lymphocytes obtained in this way were washed ×3 in TC199 (Wellcome Reagents Ltd., Beckenham, England) and stored at 4°C in TC199 containing 10% heat-inactivated foetal calf serum (FCS) until use. Peritoneal exudate cells (PEC) were induced in Hartley guinea-pigs (200–400 g) by i.p. stimulation with 10 ml of liquid paraffin, and collected and processed as described previously (Rees and Potter, 1973).

Lymphocytes and PEC were mixed to give a final concentration of $2 \cdot 0 \times 10^6$ and $1 \cdot 0 \times 10^7$ cells/ml respectively in TC199. This suspension was drawn into 10μl microcaps (Drummond Scientific Co., USA.) and sealed at one end with Cristaseal (Hawksley and Sons Ltd., Lancing, England) and centrifuged at 1500 *g* for 5 min. The tubes were cut at the cell–fluid interface and fixed in the appropriate wells of a migration plate (Sterilin Ltd., Teddington, England) with a spot of silicone grease. Incubations were conducted in TC199 containing 10% heat-inactivated FCS and EF at a concentration of 100 μg/ml. Duplicate control wells were set up without EF. Two wells each containing 3 capillary tubes were set up for each treatment. Every well was sealed with a glass coverslip fixed with silicone grease and incubated at 37°C after gassing with a mixture of 5%$CO_2$/95% air. The migration fans were drawn after 24h incubation, with the aid of a projection microscope, and the areas measured by planimetry. The percentage inhibition of macrophage migration with EF was calculated from the following formula:

$$\% \text{ inhibition} = 100 \times \left[1 - \frac{\text{Area of migration with EF}}{\text{Area of migration without EF}}\right]$$

The significance of migration inhibition was assessed using Student's *t* test. A level of $P < 0 \cdot 01$ was considered as significant.

TABLE I.—*MMI by Lymphocytes from Cancer Patients* in the Presence of Encephalitogenic Factor*

| Disease stage | No. tested | No. (%) showing significant MMI ($P < 0 \cdot 01$) |
|---|---|---|
| I | 25 | 16(64) |
| II | 17 | 12(70) |
| III | 28 | 16(57) |
| Total | 70 | 44(63) |

* 16 carcinomas of the lung, 4 carcinomas of the larynx, 14 carcinomas of the breast, 7 carcinomas of the cervix, 4 lymphomas, 3 basal-cell carcinomas, 5 carcionomas of the bladder, 2 carcinomas of the ovary, 2 malignant melanomas, 2 carcinomas of the endometrium, 1 carcinoma of the tongue, 1 carcinoma of the stomach, 1 carcinoma of the thyroid, 1 carcinoma of the lip, 1 carcinoma of the rectum, 1 carcinoma of the oesophagus, 1 carcinoma of the vagina-urethra, 1 carcinoma of the ethmoid, 1 lymphosarcoma, 1 prostatic sarcoma and 1 leiomyosarcoma of the jejunum.

*Serum inhibition of EF-mediated migration inhibition.*—Ten ml of venous blood collected from each individual under test was allowed to clot at room temperature for 1 h and was centrifuged at 1500 *g* for 10 min. The serum was harvested from the tube and heat-inactivated at 56°C for 30 min. The effects of autologous serum drawn from the same patient, or homologous serum drawn from different patients with the same or different tumour types, upon EF-mediated migration inhibition were studied by including the serum under investigation in a duplicate set of wells at a 10% concentration with and without EF.

### RESULTS

### *Sensitization to EF in cancer patients and controls*

A total of 70 individuals with malignant disease were tested for sensitivity to EF with the MMI test. The results are shown in Table I and in Fig. 1. A significant inhibition of macrophage migration ($P < 0 \cdot 01$) with EF was seen in 44 of these patients. Division of this group into 3 clinical stages (I–III) showed that the frequency of sensitivity to EF was about the same in all 3 (Table I). Patients with non-malignant diseases were divided into individuals with chronic bronchitis, warts, multiple sclerosis, neurological conditions and others. The results for 34 individuals tested with non-malignant disease are shown in Table II and Fig. 2. Significant migration inhibition with EF was seen in 11 of these patients, the highest frequency of sensitivity being observed in 9 patients with various neurological conditions, among whom 4 showed sensitivity, and the lowest frequency in a group of 6 individuals with simple epidermal warts, among whom one showed significant migration inhibition. Lymphocytes from 18 healthy individuals were also tested for sensitivity to EF with the MMI test. The results are shown in Table II and in Fig. 2. Of the 18 tested, one showed significant migration inhibition with EF.

### *Effects of autologous serum*

The effects of autologous serum included

FIG. 1.—Percentage inhibition of macrophage migration (MMI) by lymphocytes from cancer patients in the presence of encephalitogenic factor.

in a duplicate set of control and EF-containing wells were investigated in 53 cancer patients, in 34 patients with non-malignant disease and in 18 healthy individuals. The results obtained for the 53 cancer patients tested in the absence and presence of autologous serum are shown in

FIG. 2.—Percentage inhibition of macrophage migration (MMI) by lymphocytes from patients with non-malignant disease and from healthy individuals in the presence of encephalitogenic factor.

† 3 patients with systemic lupus erythematosus and one patient with polymyositis.

TABLE II.—*MMI by Lymphocytes from Patients with Non-malignant Diseases and from Healthy Individuals in the Presence of Encephalitogenic Factor*

| Diagnosis | No. tested | No. (%) showing significant MMI ($P<0\cdot01$) |
|---|---|---|
| Warts | 6 | 1(17) |
| Chronic bronchitis | 7 | 2(28) |
| Disseminated sclerosis | 8 | 3(37) |
| Neurological conditions* | 9 | 4(44) |
| Others† | 4 | 1(25) |
| Total | 34 | 11(32) |
| Healthy individuals | 18 | 1(6) |

* 2 Parkinson's disease, 3 cerebral vascular accidents, 1 encephalopathy, 1 peripheral neuropathy (cause unknown), 1 weakness of the lower limbs (cause unknown) and 1 brain-stem lesion.

† 1 polymyositis and 3 systemic lupus erythematosus.

TABLE III.—*MMI by Lymphocytes from Cancer Patients* in the Presence of Encephalitogenic Factor, with and without Autologous Serum*

| Disease stage | No. tested | No. (%) showing significant MMI ($P<0\cdot01$): Without serum | With autologous serum |
|---|---|---|---|
| I | 17 | 9(53) | 2(12) |
| II | 14 | 9(64) | 3(21) |
| III | 22 | 13(59) | 4(18) |
| Total | 53 | 31(58) | 9(17) |

* 11 carcinomas of the lung, 9 carcinomas of the breast, 7 carcinomas of the cervix, 3 carcinomas of the bladder, 2 carcinomas of the ovary, 2 basal cell carcinomas, 2 carcinomas of the larynx, 2 malignant melanomas, 3 lymphomas, 1 carcinoma of the tongue, 1 carcinoma of the endometrium, 1 carcinoma of the rectum, 1 carcinoma of the lip, 1 carcinoma of the thyroid, 1 carcinoma of the stomach, 1 carcinoma of the vagina–urethra, 1 carcinoma of the ethmoid, 1 carcinoma of the oesophagus, 1 leiomyosarcoma of the jejunum, 1 prostatic sarcoma and 1 lymphosarcoma.

TABLE IV.—*MMI by Lymphocytes from Patients with Non-maligant Diseases and from Healthy Individuals in the Presence of Encephalitogenic Factor, with and without Autologous Serum*

| Diagnosis | No. tested | No. (%) showing significant MMI ($P<0\cdot01$): Without serum | With autologous serum |
|---|---|---|---|
| Warts | 6 | 1(17) | 0 |
| Chronic bronchitis | 7 | 2(28) | 2(28) |
| Disseminated sclerosis | 8 | 3(37) | 3(37) |
| Neurological conditions* | 9 | 4(44) | 2(22) |
| Others* | 4 | 1(25) | 0 |
| Total | 34 | 11(32) | 7(20) |
| Healthy individuals | 18 | 1(5) | 1(5) |

* See Table II for details.

TABLE V.—*The Effects of Homologous Serum on MMI by Lymphocytes from Patients with Various Tumour Types*

| | | No. showing significant MMI ($P<0\cdot01$) | | | | | |
|---|---|---|---|---|---|---|---|
| | | | Homologous serum with (+) or without (−) autologous blocking from: | | | | |
| | | | | Carcinoma lung | | Carcinoma breast | |
| Lymphocytes from | No. tested | Without serum | Normal | − | + | − | + |
| Carcinoma breast | 4 | 1 | 1 | 1 | 0 | 1 | 0 |
| Carcinoma lung | 3 | 3 | 3 | 3 | 0 | 2 | 0 |
| Carcinoma larynx | 1 | 1 | 1 | 1 | 0 | 1 | 0 |
| Carcinoma bladder | 2 | 2 | 2 | 2 | 0 | 2 | 1 |
| Basal-cell carcinoma | 1 | 1 | 1 | 1 | 0 | 1 | 1 |
| Total | 11 | 8 | 8 | 8 | 0 | 7 | 2 |

Table III. Of these, 31 patients showed significant migration inhibition with EF in the absence of autlogous serum, but this was abolished in 22 of them in the presence of autologous serum. Division of the cancer patients into 3 clinical stages (I–III) did not show any significant difference in frequency of serum-blocking between the three stages.

Autologous serum from 34 individuals with various non-malignant conditions blocked EF-mediated migration inhibition in 4/11 patients showing significant migration inhibition with EF (Table IV). Serum-blocking activity in this group occurred in 2 patients with Parkinson's disease, one patient with warts and one with systemic lupus erythematosus.

Autologous serum from healthy individuals produced no significant change in macrophage migration from that in the absence of autlogous serum (Table IV).

*Effects of homologous serum*

Serum from a healthy individual, 2 patients with carcinoma of the lung (one with and one without autologous blocking activity) and 2 patients with carcinoma of the breast (one with and one without autologous blocking activity) were tested against lymphocytes from 4 patients with carcinoma of the breast, 3 with carcinoma of the lung, 2 with carcinoma of the bladder, 1 with carcinoma of the larynx and 1 with a basal-cell carcinoma of the scalp, for evidence of blocking activity by homologous serum. The results are shown in Table V. Of the 11 cancer patients with the MMI test in the absence of serum, 8 showed significant migration inhibition with EF. In the presence of normal serum, all 8 retained significant migration inhibition with EF. When serum from a patient with carcinoma of the lung without autologous blocking activity was used, all 8 patients retained demonstrable sensitivity. However, serum from a carcinoma of the lung *with* autologous blocking activity abolished EF-mediated migration inhibition in all 8 patients, regardless of the tumour type from which the lymphocytes under test were derived (Table V). Serum from a carcinoma of the breast without autologous blocking activity abolished EF-mediated migration inhibition in one patient with a carcinoma of the lung, whilst serum from a carcinoma of the breast *with* autologous blocking activity abolished EF-mediated migration inhibition in 6/8 sensitized individuals.

### DISCUSSION

The results of the present study for lymphocyte sensitivity to EF in malignant and non-malignant disease agree well with those of other workers using the same techniques (Shelton *et al.*, 1975; Light *et al.*, 1975; Singer *et al.*, 1975). However, the observed frequency of sensitivity in cancer is lower with the MMI test than in the results reported for the MEM test (Field and Caspary, 1970; Caspary and Field, 1971). This may reflect differences in sensitivity between the two techniques, and this has been discussed previously (Hughes and Paty, 1971; Shelton *et al.*, 1975). The frequency of sensitivity to EF in the present study was similar in all 3 clinical stages of cancer, and it is thus not possible to discriminate between patients with small primary tumours and extensive metastatic disease by the MMI test. However, Field and Caspary (1972) found a lower degree of lymphocyte sensitivity to CaBP in patients with advanced malignant disease, using a standard number of lymphocytes in the MEM test. This effect was abolished by increasing the number of lymphocytes under test 5- or 10-fold. Thus the inability of the MMI test to discriminate between early and advanced malignant disease may be due to the greater number of lymphocytes routinely used in the test ($2{\cdot}0\times10^6$ lymphocytes as opposed to $0{\cdot}5\times10^6$ in the MEM test).

The present study has also confirmed the findings of Shelton *et al.* (1975) that sensitivity to EF also occurs in a number of individuals with non-malignant conditions. The high level of sensitization seen in this study in patients with neurological conditions suggests that conditions that might involve nervous parenchymal destruction, result in the release of the EF molecule from the intraperiod line of lamellar myelin (Dickinson *et al.*, 1970) and subsequent sensitization. Alternatively, EF sensitivity may prove to be a primary factor in some disease processes. For instance, the histological appearance of the lesions found in the central nervous system of patients with multiple sclerosis suggests an active immunological process (Nilsson, 1972). Indeed it may be that a proportion of the carcinomatous neuropathies, sometimes associated with demyelination (Schlaepfer, 1974) observed in a small number of cancer patients, may be a secondary pathological process brought about by the generation of autoaggressive lymphocytes by neoantigen(s) on the tumour-cell surface and immunologically cross-reactive with EF or an EF-like molecule in the central nervous system.

Abolition of EF-mediated migration inhibition by autologous serum was seen in a large proportion of the cancer patients studied, and occurred with a similar frequency in all 3 clinical stages. Moreover, serum which abolished the lymphocyte response to EF in the autologous situation also abolished the EF response of lymphocytes from patients with different tumour types. Serum abolition of the lymphocyte response to EF was also seen in 4/11 sensitized individuals with non-malignant conditions. This serum effect thus differs markedly from serum blocking phenomena observed in cancer patients and thought to be mediated by free circulating antigen or antigen–antibody complexes (Currie and Basham, 1972; Currie, 1973; Sjögren *et al.*, 1971). Here, serum blocking activity correlates well with the extent of the disease (Currie, 1973; Bray and Holt, 1975) the highest titres being found in advanced disease, and also proves to be specific for the tumour type from which the serum was derived (Hellström *et al.*, 1971). It seems likely that the serum effect seen in the present study might be closely akin to the serum lymphocyte-depressive factor described by Field and Caspary (1972) and thought to be due to the $alpha_2$ macroglobulin component of serum (Ford, Caspary and Shenton, 1973).

In conclusion, it thus seems that the MMI test in its present form, used to measure EF sensitivity and associated serum inhibitory activity, is unable to discriminate between patients with early and advanced malignant disease.

We would like to thank the medical and nursing staff of Weston Park Hospital, the Hallamshire Hospital and the Royal Hospital, Sheffield, for their assistance and continued advice. This work was supported by a grant from the Yorkshire branch of the Cancer Research Campaign.

### REFERENCES

Bray, A. E. & Holt, P. G. (1975) Serum Blocking Factor as an Index of Metastatic Spread Following Primary Tumour Excision. *Eur. J. Cancer*, **11**, 855.

Carnegie, P. R., Caspary, E. A. & Field, E. J. (1973) Isolation of an "Antigen" from Malignant Tumours. *Br. J. Cancer*, **28**, Suppl. 1, 219.

Caspary, E. A. & Field, E. J. (1971) Specific Lymphocyte Sensitization in Cancer: Is there a Common Antigen in Human Malignant Neoplasia? *Br. med. J.*, ii, 613.

Coates, A. S. & Carnegie, P. R. (1975) Immunological Cross Reactivity between Basic Proteins of Myelin and Cancer. 1. Lymphocyte Transformation Studies in Immunized Guinea Pigs. *Clin. exp. Immun.*, **22**, 16.

Currie, G. (1973) The Role of Circulating Antigen as an Inhibitor of Tumour Immunity in Man. *Br. J. Cancer*, **28**, Suppl. 1, 153.

Currie, G. & Basham, C. (1972) Serum Mediated Inhibition of the Immunological Reactions of the Patient to his own Tumour: A Possible Role for Circulating Antigen. *Br. J. Cancer*, **26**, 427.

Dickinson, J. P., Caspary, E. A. & Field, E. J. (1973) A Common Tumour Specific Antigen. 1. Restriction *In vivo* to Malignant Neoplastic Tissue. *Br. J. Cancer*, **27**, 99.

Dickinson, J. P., Jones, K., Aparicio, S. & Lumsden, C. E. (1970) Localization of Encephalitogenic Basic Protein in the Intraperiod Line of Lamellar Myelin. *Nature, Lond.*, **227**, 1133.

Field, E. J. & Caspary, E. A. (1970) Lymphocyte Sensitization: An *In vitro* Test for Cancer. *Lancet*, ii, 1337.

Field, E. J. & Caspary, E. A. (1972) Lymphocyte Sensitization in Advanced Malignant Disease: a Study of Serum Lymphocyte Depressive Factor. *Br. J. Cancer*, **26**, 164.

Ford, W. H., Caspray, E. A. & Shenton, B. (1973) Purification and Properties of a Lymphocyte Inhibition Factor from Human Serum. *Clin. exp. Immun.*, **15**, 169.

Goldstone, A. H., Kerr, L. & Irvine, W. J. (1973) The Macrophage Electrophoretic Mobility Test in Cancer. *Clin. exp. Immun.*, **14**, 469.

Hellström, I., Sjögren, H. O., Warner, G. & Hellström, K. E. (1971) Blocking of Cell Mediated Tumour Immunity by Sera from Patients with Growing Neoplasms. *Int. J. Cancer*, **7**, 226.

Hughes, D. & Paty, D. W. (1971) Lymphocyte Sensitivity in Cancer. *Br. med. J.*, ii, 770.

Light, P. A., Preece, A. W. & Waldron, H. A. (1975) Studies with the Macrophage Migration Inhibition Test in Patients with Malignant Disease. *Clin. exp. Immun.*, **22**, 279.

McDermott, J. R., Caspary, E. A. & Dickinson, J. P. (1974) Antigen Cross Reactivity in the Macrophage Electrophoretic Mobility Test. A Study Using Cellular Affinity Chromatography. *Clin. exp. Immun.*, **17**, 103.

Mitchell, H. (1973) Structural Conformation of Tumour Antigen. *Lancet*, ii, 1061.

Nilsson, O. (1972) Immunological Aspects of Demyelinating Diseases. *Acta. neurol. scand.*, **48**, Suppl. 51, 321.

Pritchard, J. A. V., Moore, J. L., Sutherland, W. H. & Joslin, C. A. F. (1973) Technical Aspects of the Macrophage Electrophoretic Mobility

(MEM) Test for Malignant Disease. *Br. J. Cancer*, **28**, Suppl. 1, 229.

REES, R. C. & POTTER, C. W. (1973) Immune Response to Adenovirus 12-induced Antigens as measured *In vitro* by the Macrophage Migration Inhibition Test. *Eur. J. Cancer*, **9**, 497.

SCHLAEPFER, W. W. (1974) Axonal Degeneration in the Sural Nerves of Cancer Patients. *Cancer, N.Y.*, **34**, 371.

SHELTON, J. B., POTTER, C. W. & CARR, I. (1975) Cellular Immunity to Myelin Basic Protein in Man and in Animal Model Systems as Measured by the Macrophage Migration Inhibition Test. *Br. J. Cancer*, **31**, 528.

SINGER, A., SHELTON, J., HILL, S. & POTTER, C. (1975) Cellular Immunity to Myelin Basic Protein in Women with Dysplasia and Carcinoma *In situ* of the Cervix. *Br. J. Obstet. Gynaecol.*, **82**, 820.

SJÖGREN, H. O., HELLSTRÖM, I., BANSAL, S. C. & HELLSTRÖM, K. E. (1971) Suggestive Evidence that the Blocking Antibodies of Tumour Bearing Individuals may be Antigen–antibody Complexes. *Proc. natn. Acad. Sci. USA*, **68**, 1372.